# Tribute to John C. Martin at the Twentieth Anniversary of the Breakthrough of Tenofovir in the Treatment of HIV Infections

**DOI:** 10.3390/v13122410

**Published:** 2021-12-02

**Authors:** Erik De Clercq

**Affiliations:** Department of Microbiology, Immunology and Transplantation, Rega Institute for Medical Research, Katholieke Universiteit Leuven, 3000 Leuven, Belgium; erik.declercq@kuleuven.be

**Keywords:** HIV, CMV, tenofovir, TDF, TAF

## Abstract

At Bristol-Myers (BM) (1985–1990), John C. Martin started his HIV career with directing the clinical development of didanosine (ddI) and stavudine (d4T). During this period, he became aware of the acyclic nucleoside phosphonates (ANPs), such as (*S*)-HPMPA and PMEA, as potential antiviral drugs. Under his impulse, BM got involved in the evaluation of these ANPs, but the merger of BM with Squibb (to become BMS) incited John to leave BM and join Gilead Sciences, and the portfolio of the ANPs followed the transition. At Gilead, John succeeded in obtaining the approval from the US FDA for the use of cidofovir in the treatment of cytomegalovirus (CMV) retinitis in AIDS patients, which was reminiscent of John’s first experience with ganciclovir (at Syntex) as an anti-CMV agent. At Gilead, John would then engineer the development of tenofovir, first as TDF (tenofovir disoproxil fumarate) and then as TAF (tenofovir alafenamide) and various combinations thereof, for the treatment of HIV infections (*i*), TDF and TAF for the treatment of hepatitis B (HBV) infections (*ii*), and TDF and TAF in combination with emtricitabine for the prophylaxis of HIV infections (*iii*).

## 1. Prologue

On 26 October 2001, 20 years ago, tenofovir in its prodrug form, TDF (tenofovir disoproxil fumarate), was formally approved by the US FDA (Food and Drug Administration) for the treatment of HIV infections. John C. Martin, former President and Chief Executive Officer (1995–2020) of Gilead Sciences, intended to commemorate the 20th anniversary of this milestone event in the fight against AIDS (HIV infection), but John suddenly died on 30 March 2021, a few weeks before his 70th birthday.

## 2. Introduction to the Antiviral Drug Field: Ganciclovir

John C. Martin started his scientific career at Syntex (now incorporated in Roche) and he is co-inventor (together with Julien Verheyden) of ganciclovir, then named 9-(1,3-dihydroxy-2-propoxymethyl) guanine DHPG [1] (US patent 4 355 032). Ganciclovir was discovered shortly after acyclovir and has been further documented in several studies.

Ganciclovir would later be derivatized to its aminoacyl ester, valganciclovir, in analogy with acyclovir that was converted to valacyclovir, its valine ester, to ensure improved oral bioavailability. In contrast with acyclovir and valacyclovir, ganciclovir and valganciclovir proved effective against cytomegalovirus (CMV) and this explains why it was specifically pursued for the treatment of CMV infections, although it had also marked activity against herpes simplex virus (HSV) infections.

### 2.1. Switch to the Anti-HIV Drug Field: Didanosine

John then moved to Bristol-Myers (BM) and this also meant a shift from the herpesvirus field to the field of human immunodeficiency virus (HIV) which, until 1987, had been given different names, including HTLV-III and LAV. Didanosine or 2′,3′-dideoxyinosine (ddI) has been described by Mitsuya and Broder [2], together with various other 2′3′-dideoxynucleoside analogues, as inhibitors of HTLV-III, and ddI would be selected at BM for further clinical development. It was approved by the US FDA on 9 October 1991 and is still used clinically for the treatment of HIV infections [3,4,5].

### 2.2. 2′,3′-Didehydro-2′,3′-dideoxythymidine (Stavudine, d4T)

At BM, John directed the clinical development of ddI and, thereafter, that of d4T. How the latter fared is further described by Martin et al. [6]. The discovery of the anti-HIV activity of d4T (then called 2′,3′-dideoxythymidinene) was first described in January 1987 by Masanori Baba et al. [7] and confirmed half a year later by Hamamoto et al. [8] and Lin et al. [9]. For a certain number of years, stavudine (Zerit^®^) has been globally the most prescribed anti-HIV drug [10,11,12].

### 2.3. The Breakthrough of the ANPs (Acyclic Nucleoside Phosphonates)

**Figure 1:**
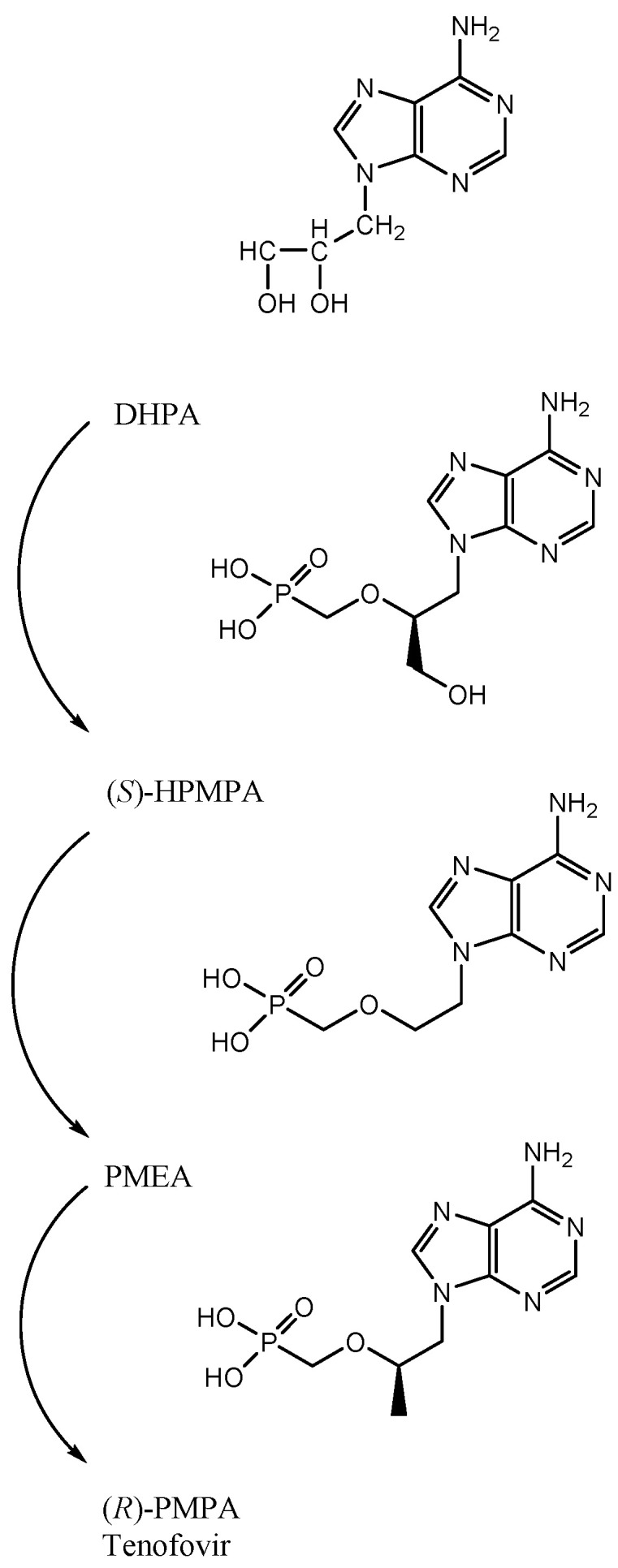
Genealogy of tenofovir.

The advent of (*S*)-HPMPA (*S*-9-(3-hydroxypropyl-2-methoxy) phosphonyl adenine) (Figure 1) heralded the birth of a new class of antiviral agents, that of the acyclic nucleoside phosphonates (ANPs), with a broad-spectrum activity against DNA viruses and retroviruses, including HIV [13,14]. In contrast with acyclovir, which only showed activity against herpesviruses encoding a specific thymidine kinase, (*S*)-HPMPA proved active against all sorts of herpesviruses, pox-, adeno-, and papillomaviruses. Based on preliminary toxicity studies, preference was given to its cytosine counterpart, *S*-1-(3-hydroxypropyl)-2-methoxy) cytosine ((*S*)-HPMPC), for further clinical development. Yet, anecdotal data (unpublished) point to the clinical usefulness of topical (*S*)-HPMPA in the treatment of ocular adenovirus infections.

## 3. PMEA: 9-(2-Methoxyethyl) Adenine (Adefovir)

**Figure 2:**
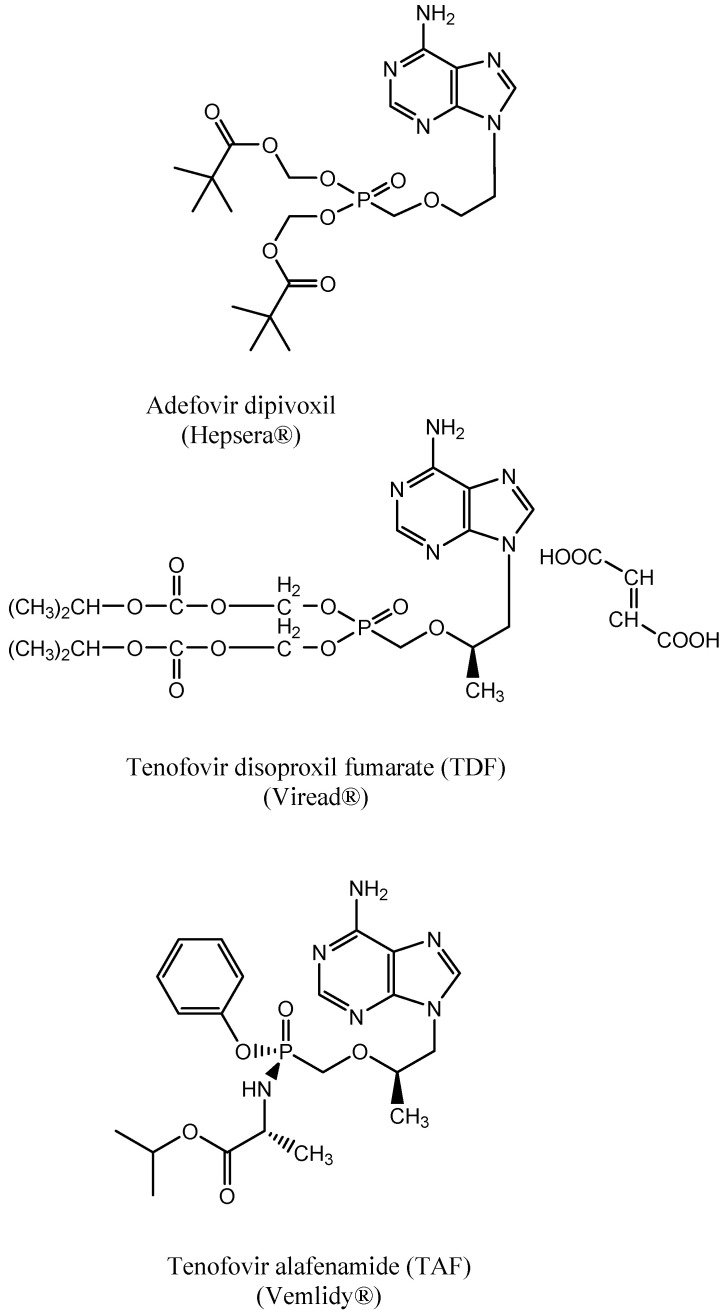
Prodrugs of PMEA (adefovir) and (*R*)-PMPA (tenofovir).

Simultaneously with (S)-HPMPA, a simpler, not-racemic compound, PMEA, was described as a potential anti-retroviral drug [13]. It was also evaluated for this purpose [15,16]. Although adefovir was primarily pursued as its prodrug, adefovir dipivoxil (to ensure oral bioavailability, Figure 2), for the treatment of HIV infections, it was also shown to be active against hepatitis B virus (HBV) [17,18,19,20]. Adefovir dipivoxil was eventually not pursued for the treatment of HIV infections because of two reasons; (*i*) at the required dosage (62.5 mg p.o. daily), it was estimated to be nephrotoxic and (*ii*) a new ANP, (R)-PMPA, had in the meantime been developed that could be administered without toxicity at a much higher dosage (300 mg). Furthermore, for the treatment of HBV infections, adefovir dipivoxil could be administered, without risk of nephrotoxicity, at a much lower dose (10 mg p.o. daily) and, hence, adefovir dipivoxil was marketed as Hepsera^®^ for this indication (FDA approval followed in 2002). Its efficacy in the treatment of HBV infections was clearly shown by Marcellin et al. [21] and Hadziyannis et al. [22,23].

## 4. *S*-1-(3-Hydroxypropyl-2-methyphosphonyl) Cytosine (Cidofovir)

While (*S*)-HPMPA was not further clinically developed by Gilead Sciences, its cytosine counterpart was, effectively so, and, under the direct guidance of John, it acquired FDA approval in 1996 (as the first ANP in history) for the treatment of CMV retinitis in AIDS patients, a severe complication of AIDS leading to blindness that is no longer observed nowadays thanks to the successful use of the anti-HIV drugs in the treatment of AIDS. It is remarkable that John’s first compound at Gilead Sciences (which he had joined in 1991) was for CMV, the same virus with which he had started his career at Syntex. That cidofovir, marketed as Vistide^®^, could be an effective agent for the treatment of CMV infections was demonstrated in 1988 by Snoeck et al. [24]. Its clinical usefulness for the treatment of CMV retinitis in AIDS patients was documented by the investigations of Lalezari et al. [25,26,27,28]. Cidofovir has also been used on a compassionate base in the treatment of various other DNA viruses (i.e., HPV (human papilloma virus)) infections [29]. It would also be efficacious in the treatment of poxvirus infections (i.e., monkeypox) [30].

## 5. First Description of (*R*)-PMPA, Later Named Tenofovir

Following the description of the racemic mixture (*RS*)-FPMPA (*RS*-9-(3-fluoropropyl-2-methoxyphosphonyl) adenine) and its anti-retrovirus properties [31], Antonín Holý focused on the synthesis of (*R*)- and (*S*)-9-(3-propyl-2-methoxyphosphonyl) adenine ((*R*)-and (*S*)-PMPA), as well as their diaminopurine derivatives. (*R*)-PMPA was found to inhibit the replication of murine retroviruses and HIV [32], the diaminopurine derivative (*R*)-PMP DAP being more potent than (*R*)-PMPA. Yet, (*R*)-PMPA was selected for further development, the reasoning of John being that nature had selected the 6-aminopurine (adenine) as the heterocyclic base, and there must have been a rationale for this choice, putatively mutagenicity. Anyway, (*R*)-PMPA became the clinical drug candidate (please note that (*R*) has the same configuration as (*S*)-HPMPA but the designation of (*S*) to (*R*) had to be adapted conform with the chirality rules of Vladimir Prelog).

In a very recent paper [33], Lilian Lou and John underscored the importance of the methyl group (hydrophobicity) in tenofovir, as originally communicated at a scientific advisory board meeting at Gilead Sciences by John Mellors.

## 6. Complete Protection of SIV Infection by (*R*)-PMPA

**Figure 3:**
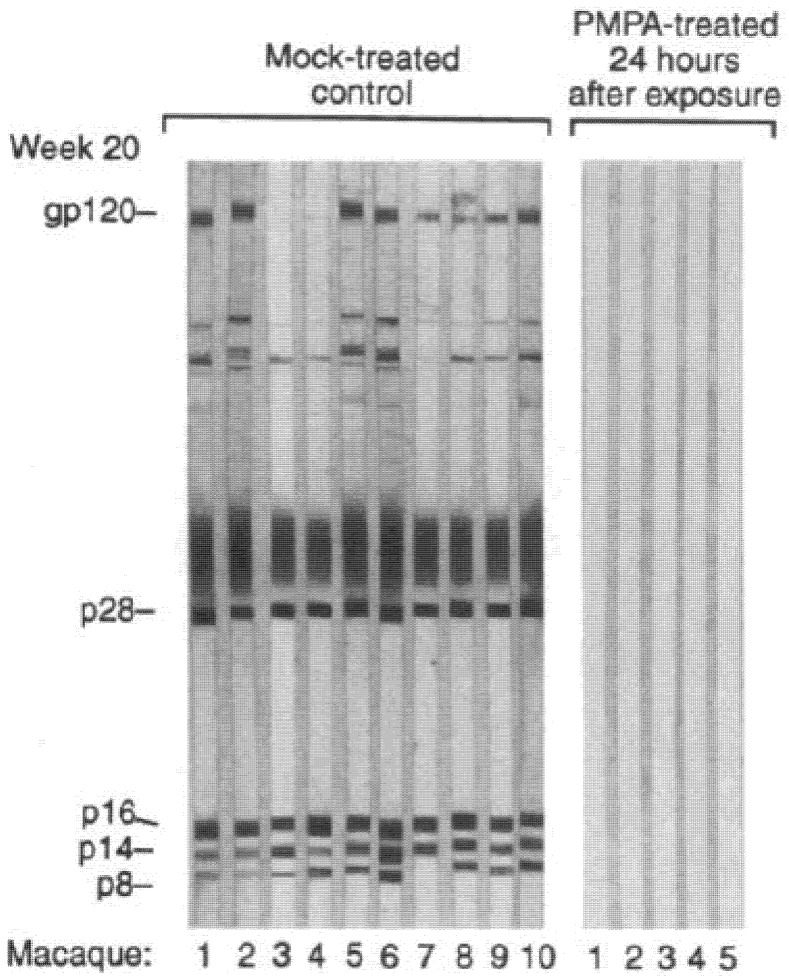
Activity of (*R*)-PMPA when injected shortly before or after SIV infection in rhesus macaques: first prediction that tenofovir may be effective in therapy and prophylaxis of HIV infection in humans (figure taken from Tsai et al. [34]).

The date of 17 November 1995 was a memorable day. On this day, the paper of Tsai et al. [34] appeared in *Science*, stating that (*R*)-PMPA conferred complete protection against simian immunodeficiency virus (SIV) in rhesus macaque monkeys when administered (subcutaneously) within a few days before or after the infection. The outcome varied from 100% protection in the five monkeys treated with (*R*)-PMPA to 0% protection in the 10 control monkeys (Figure 3). I communicated these observations on Belgian television, and the journalist responsible for the interview asked whether this meant “the morning after pill”. I replied that it was not a pill yet, let alone the morning after, but 16 years later, on 16 July 2012, the FDA approved the use of tenofovir (in combination with emtricitabine) for the prophylaxis of HIV infection. Just an additional remark: in their study, Tsai et al. had also included AZT (azidothymidine, Retrovir^®^), the first anti-HIV drug ever to be approved by the FDA in 1987 following its discovery in 1985 [35], and AZT proved much less effective than (*R*)-PMPA in suppressing SIV infection in monkeys [34].

## 7. Tenofovir Disoproxil

Like adefovir, tenofovir is only poorly bioavailable when administered by the oral route. To increase the oral bioavailability, tenofovir was converted to its prodrug, not to its bispivaloxyloxymethyl as for adefovir dipivoxil, but its bisisopropyloxycarbonyloxymethyl or disoproxil ester. This led to tenofovir disoproxil [36,37]. Several clinical studies have documented the anti-HIV efficacy of tenofovir disoproxil [38,39,40,41,42].

## 8. Tenofovir Disoproxil Fumarate (TDF)

After tenofovir disoproxil had been formulated as its fumarate, it was offered to the US FDA and approved on 26 October 2001 for the therapy of HIV infections. It was then promptly marketed as Viread^®^. Shortly thereafter (4 February 2002), it was also launched on the European market. The launching of TDF first in the US, then the EU, and finally the rest of the world, could be considered as the most important milestone marking the scientific career of John C. Martin.

## 9. TDF for the Treatment of HBV Infections

While approved in 2001 for the therapy of HIV infection, it took seven more years for the FDA to approve TDF for the treatment of HBV infections. This approval followed a comparative study of Marcellin et al. [43], showing that TDF was clearly more effective in the treatment of chronic hepatitis B (as monitored by the HBV viremia) than adefovir dipivoxil.

## 10. Truvada^®^: Combination of TDF with Emtricitabine

On 2 August 2004, the US FDA approved the combination of emtricitabine with TDF for the treatment of HIV infections. Emtricitabine (Emtriva^®^) is the (−) enantiomer of FTC ((−)FTC), which, itself, is the 5-substituted derivative of 3TC ((−)-3′-thio-2′,3′-dideoxycytidine), where the (−) designation is included in the abbreviation of 3TC while, to be chemically correct, the designation (−) should be added before FTC. Although 3TC (Epivir^®^) has been used (and approved) for the treatment of both HIV and HBV infections, it is remarkable that (−)FTC was never approved for the treatment of HBV infections and, likewise, Truvada^®^ has never been approved for the treatment of HBV infections, while it was approved on 16 July 2012 for the prophylaxis of HIV infections (see infra).

## 11. Combinations of TDF with Other Anti-HIV Drugs

Following emtricitabine, efavirenz was added to the combination with TDF, thus resulting in the triple-drug combination Atripla^®^ (TDF + emtricitabine + efavirenz), with efavirenz representing the first NNRTI (non-nucleoside reverse transcriptase inhibitor) ever entering a combination regimen for the treatment of HIV infections. Atripla^®^ was approved on 12 July 2006. This triple-drug combination was reminiscent of the triple-drug regimens routinely used in the treatment of tuberculosis (i.e., isoniazid + rifampicin + pyrazinamide or ethambutol). The rationale is to (*i*) obtain synergism between compounds interacting at different molecular targets, (*ii*) diminish toxicity by lowering the individual dosages, and (*iii*) reduce or even prevent the risk for resistance development.

With the advent of rilpivirine (Edurant^®^), efavirenz was replaced by this new NNRTI in the combination approved in 2011 (10 August 2011) and launched in the US as Complera^®^ and in the EU as Eviplera^®^. Then followed Stribild^®^ in 2012 (27 August 2012), with the first INSTI (integrase strand transfer inhibitor), elvitegravir, and a pharmaco-enhancer, cobicistat (a ritonavir analogue without anti-HIV activity), meant to enhance the plasma levels of elvitegravir.

## 12. Advent of Tenofovir Alafenamide (TAF)

In attempts to increase the uptake of tenofovir by the lymphoid T cells, the target cells for HIV replication, and thereby decrease the risk for undesirable side effects such as nephrotoxicity and bone demineralization, a new tenofovir prodrug, the proTide tenofovir alafenamide (TAF) (GS-7340) was designed [44]. TAF was, indeed, preferentially taken up by the lymphoid cells [45] and this advantage enabled a drastic reduction in the dosage of this tenofovir prodrug compared to that of TDF with, concomitantly, decreased kidney and bone toxicities. Replacement of TDF by TAF in Gilead’s portfolio on HIV treatment has been previously reviewed [46].

## 13. TAF for the Treatment of HBV Infections

As successor for TDF, TAF (Vemlidy^®^) was also approved for the treatment of HBV infections (10 November 2016), just as it had been before for the treatment of HIV infections. The rationale behind this is that TAF, akin to what has been observed for lymphoid cells, is also rapidly metabolized to its active diphosphate metabolite in the liver cells [46]. Furthermore, as has been seen with TDF, no further attempts were undertaken to clinically pursue the combination of TAF with emtricitabine for the treatment of HBV infections.

## 14. Combinations of TAF with Other Anti-HIV Drugs

Similarly to TDF, TAF was combined with several other anti-HIV drugs, i.e., emtricitabine, marketed as Descovy^®^ and approved by the FDA on 4 April 2016, emtricitabine and rilpivirine, marketed as Odefsey^®^ and approved by the FDA on 1 March 2016, emtricitabine and darunavir (Prezista, an HIV protease inhibitor), marketed as Symtuza^®^ and approved by the FDA on 17 July 2018, emtricitabine, elvitegravir and cobicistat, marketed as Genvoya^®^ and approved by the FDA on 5 November 2015, and bictegravir (an HIV integrase inhibitor like elvitegravir), marketed as Biktarvy^®^ and approved by the FDA on 7 February 2018. All these combinations are intended for once-daily oral administration, thus ensuring long-term suppression of the HIV replication.

## 15. TDF and TAF as Part of PrEP (Pre-Exposure Prophylaxis)

The combination TDF + emtricitabine, which had been approved in 2004 and since then marketed as Truvada^®^ for the treatment of HIV infections, was the first chemical ever approved by the US FDA for the prophylaxis (PrEP) of HIV infections. After TDF had been substituted by TAF in the treatment of HIV infections (Descovy^®^), the latter was also allowed for PrEP (3 October 2019). PrEP with either Truvada^®^ or Descovy^®^ is meant to prevent HIV infection by those that are at risk to be infected with HIV, but PrEP obviously does not prevent infections by microorganisms other than HIV, such as chlamydia. Although not formally approved for this purpose, HBV infections could also be prevented by PrEP with either Truvada^®^ or Descovy^®^.

## 16. Total Number of Tenofovir Preparations Now on the Market

**Figure 4:**
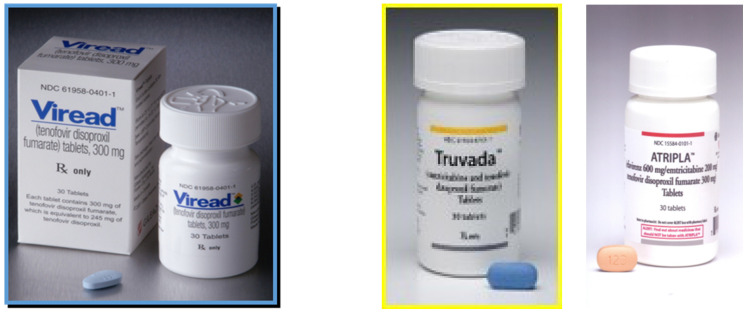

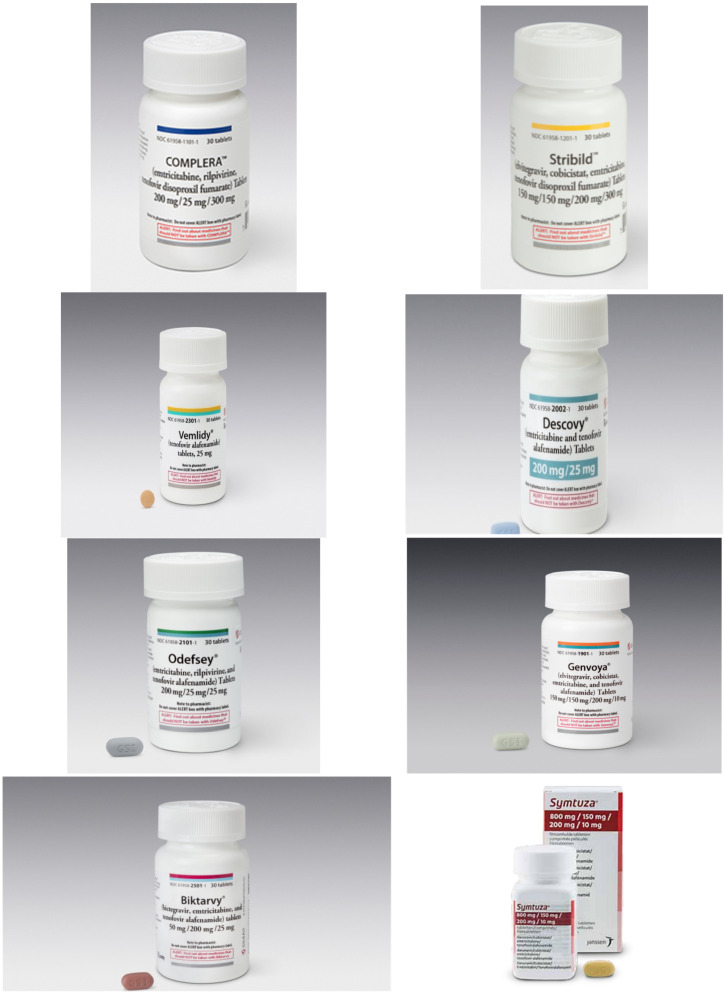

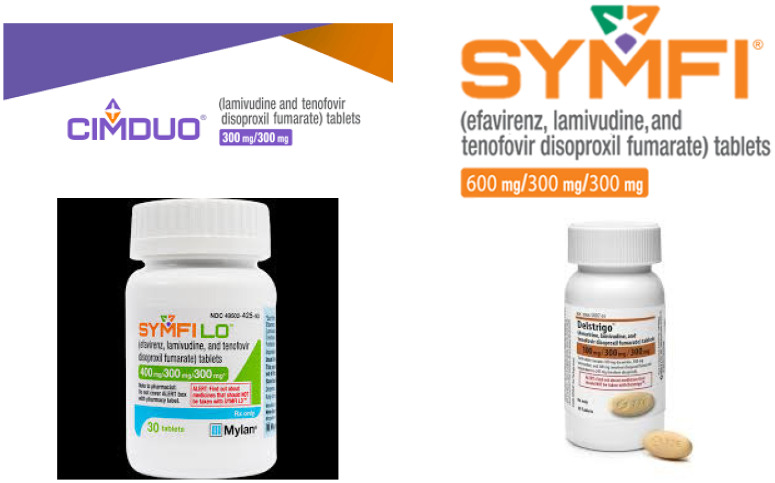
Overview of TDF- or TAF-containing combinations currently on the market.

As of today, there are now 15 preparations [47] on the market that contain tenofovir as the active ingredient (Figure 4).
TDF:Tenofovir disoproxil fumarate:Viread^®^TDF+Emtricitabine (Emtriva^®^):Truvada^®^TDF+Emtricitabine+Efavirenz (Sustiva^®^, Stocrin^®^):Atripla^®^TDF+Emtricitabine+Rilpivirine (Edurant^®^):Complera^®^, Eviplera^®^TDF+Emtricitabine
Elvitegravir+Cobicistat:Stribild^®^TAF:Tenofovir alafenamide



:Vemlidy^®^TAF+Emtricitabine



:Descovy^®^TAF+Emtricitabine+Rilpivirine

:Odefsey^®^TAF+Emtricitabine+Elvitegravir+Cobicistat:Genvoya^®^TAF+Emtricitabine+Bictegravir

:Biktarvy^®^TAF+Emtricitabine+Darunavir+Cobicistat:Symtuza^®^TDF+Lamivudine

:Cimduo™TDF+Lamivudine+Efavirenz (600 mg):Symfi™TDF+Lamivudine+Efavirenz (400 mg):Symfi Lo™TDF+Lamivudine+Doravirine:Delstrigo™

## 17. Compounds in the Pipeline

**Figure 5:**
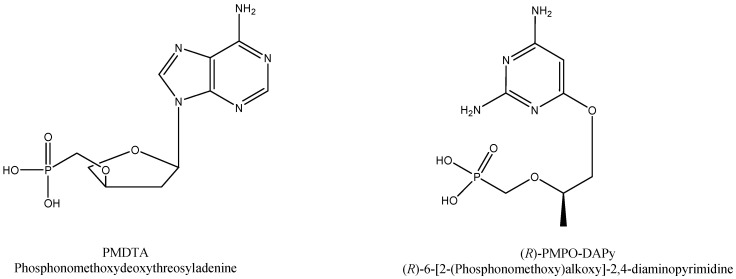
Examples of tenofovir ((*R*)-PMPA) derivatives in the pipeline (not (yet) developed clinically), as potential HIV and/or HBV inhibitors.

There are still several compounds, both cyclic and acyclic nucleoside phosphonate analogues, that have not been sufficiently explored for their potential in the treatment (or prophylaxis) of infections with retroviruses (HIV), HBV, or other DNA viruses. These include the deoxythreosyl phosphonate nucleosides PMDTA and PMDTT [48] and the 6-[2-(phosphonomethoxy)alkoxy]-2,4-diamino-pyrimidines such as PMEO-PAPy, (*R*)-HPMPO-DAPy, and (*R*)-PMPO-DAPy [49], again emphasized in a more recent overview (Figure 5) [50]. It would remain worth exploring whether any of these compounds or prodrugs derived thereof may show any advantage over existing drugs in terms of the antiviral activity spectrum, efficacy or safety, or pharmacokinetics.

## 18. Epilogue

With the sudden and unexpected death of John on 30 March 2021, we have lost the second member (Dr. Antonín Holý already died on 16 July 2012) of what could have been called, in Roman times, a triumvirate, and which I called, after seeing the magnificent monument of the Holy Trinity in Olomouc (Czech Republic), the “Holý Trinity”. In her splendid treatise on the collaboration between three scientists stemming from three parts of the world, the East (Czechoslovakia: A. Holý), the West (Belgium: myself), later to be extended to the US (John C. Martin), Mrs. Renilde Loeckx, former Ambassador of Belgium in the Czech Republic, referred to a triangle interaction that had originated during the Cold War period.

## Data Availability

Not applicable.
